# Hearing and hearing rehabilitation after obliteration of troublesome mastoid cavities

**DOI:** 10.1007/s00405-020-06041-4

**Published:** 2020-05-22

**Authors:** Simon Geerse, Tim J. M. Bost, Samira Allagul, Maarten J. F. de Wolf, Fenna A. Ebbens, Erik van Spronsen

**Affiliations:** 1 Department of Otorhinolaryngology, Amsterdam University Medical Centers, Location AMC, Amsterdam, The Netherlands; 2 Department of Otorhinolaryngology, Clinical and Experimental Audiology, Amsterdam University Medical Centers, Location AMC, Amsterdam, The Netherlands

**Keywords:** Audiometry, Pure-tone, Ear disease/surgery*, Hearing aids*, Mastoid/surgery*, Reoperation

## Abstract

**Purpose:**

The purpose of this study is the evaluation of post-operative hearing threshold after revision surgery and obliteration of troublesome canal wall down mastoidectomy cavities (CWDMCs). The ability to use and tolerate conventional hearing aids (CHAs) was also evaluated.

**Methods:**

A retrospective chart analysis of 249 patients with chronically draining CWDMCs who underwent revision surgery including obliteration of the mastoid cavity between 2007 and 2017 at the AMC location of the Amsterdam University Medical Centers (Amsterdam UMC) was performed. Patient characteristics, pre- and post-operative Merchant grade, surgical outcomes, pre- and post-operative hearing thresholds, and the ability/necessity to use a CHA or the ability/necessity to use a Bone Conduction Device (BCD) were recorded.

**Results:**

Dry ears were found in 95% of the total cohort. Residual disease was detected in 1.6% during MRI follow-up with no residual cholesteatoma in the obliterated area. In 3.2% of the patients, recurrent disease was found. A significant improvement in mean air conduction level, mean bone conduction level, and mean air-bone gap (ABG) was found post-operatively (*p* < 0.05). For all types of ossicular chain reconstruction, a significant improvement in mean Pure Tone Average was observed (*p* < 0.05). The percentage of patients with an indication for CHA was similar pre- and post-operatively (67% both pre- and post-operatively). The ability to use a CHA improved from 3% pre-operatively to 57% post-operatively (*p* < 0.001).

**Conclusion:**

This study shows that revision surgery and obliteration of CWDMCs enable successful CHA rehabilitation post-operatively. Upon this type of surgery, hearing thresholds improve significantly, but the need for rehabilitation with a CHA remains necessary in most cases.

## Introduction

It is well known that chronically draining and troublesome canal wall down mastoidectomy cavities (CWDMCs) have a profound negative effect on those inflicted [1–3]. Due to chronic drainage, conventional hearing aids (CHAs) are often not tolerated. Alternatives such as bone conduction devices (BCDs) and active middle ear implants (MEIs) have been propagated. However, functional hearing and speech discrimination of these devices are inferior to these criteria in CHAs [4]. Revision surgery and obliteration of CWDMCs with reconstruction of the ear canal have shown to ultimately address troublesome cavities and result in high rates of dry ears [5–11]. Most publications state that in case of hearing thresholds post-operatively, a conductive or mixed hearing loss remains present. The feasibility to rehabilitate hearing post-operatively is, therefore, important and essential when counselling and selecting patients for such surgery. Unfortunately, this topic seems to be almost completely neglected in the available literature regarding revision CWDMC surgery [12]. Tolerance of CHAs can potentially be restored after surgery [12]. We evaluated various outcomes related to hearing in subjects who underwent revision CWDMC surgery including obliteration of the mastoid cavity to determine two relevant topics: first, which hearing thresholds are achieved after surgery; second, to which extent is successful CHA rehabilitation and tolerance achieved post-operatively.

## Material and methods

### Participants and disease control

A retrospective chart analysis was performed of all patients with troublesome CWDMCs who underwent revision mastoid cavity surgery with partial obliteration of the mastoid and reconstruction of the ear canal at the AMC location of the Department of Otolaryngology of the Amsterdam University Medical Centers, between 2007 and 2017. Operations were performed by six different surgeons. A more detailed description of this technique has been published previously [11]. In brief, it comprises the complete removal of all remaining air cell tracts lateral to the labyrinth followed by the obliteration of the mastoid bowl with hydroxyapatite granules. The posterior ear canal wall is reconstructed with cartilage and a midtemporal artery flap. We used the Merchant classification to classify the severity of pre- and post-operative discharge [13]. We defined Merchant classification 0 and 1 as dry ears, and types 2 and 3 as wet ears. Magnetic Resonance Imaging with Diffusion-Weighted Imaging (MRI-DWI) was scheduled per protocol to detect residual and recurrent cholesteatoma in the obliterated mastoid cavity. Follow-up time was defined as the time between surgery and moment of last contact with our outpatient clinic. For retrospective cohort analysis of regular care, no Institutional Review Board approval was necessary.

### Hearing threshold

Pre- and post-operative audiometric results were analysed for all patients. The thresholds of both bone and air conduction of both ipsi- and contralateral ear were reviewed. Pure Tone Averages (PTAs) at 0.5, 1, 2, and 4 kHz were used to determine the air conduction thresholds of the affected and non-affected ear. Bone conduction levels were determined using averages at 0.5, 1, and 2 kHz. In cases where one of the frequencies was not measured or no threshold was present, this was accounted for as missing data. In addition to audiometric results, data regarding type of ossicular chain reconstruction were acquired. We identified four categories: (1) no ossicular chain reconstruction, (2) Kurz Clip^®^ partial ossicular replacement prosthesis (PORP), (3) Fisch II Spandrel^®^ total ossicular replacement prosthesis (TORP), and (4) cartilage interposition on intact stapes.

### Hearing rehabilitation

The indication for a CHA was somewhat arbitrarily defined as a PTA of equal or more than 35 dB HL. This level was chosen as the Dutch reimbursement system for CHAs requires a PTA of at least 35 dB HL. Patients with hearing losses of more than 80 dB HL were assumed to be better off with a cochlear implant and, therefore, excluded from our study. A subgroup comprised of patients who had both pre- and post-operatively a CHA indication and from whom it was registered if they used a CHA pre- and post-operatively (yes, no, and why not), was created. This subgroup enabled comparison of hearing rehabilitation and CHA tolerance pre- and post-operatively. Reasons for non-usage of CHAs as well as cases with bilateral hearing rehabilitation were identified. The use of BCDs was separately evaluated in the entire cohort.

### Statistical analysis

Statistical analyses were performed in SPSS 25.0 (Chicago, IL, USA). Data are expressed as number (%) and mean (SD). The Student’s *t *test was used to determine differences between pre-operative and post-operative hearing thresholds (both overall results and ossicular chain result sub-analysis). *p* values < 0.05 were considered to be statistically significant. A violin plot was used to illustrate the change of hearing threshold within the population of all participants. A Chi-squared test was performed to determine whether a significant difference could be observed in the use of CHA pre- and post-operatively.

## Results

### Study population and disease control

A total of 249 patients were included. The male to female distribution was 148 (59%) to 101 (41%), respectively, with a median age of 45 years (ranging from 6 to 86 years). Median time to follow-up was 52 months with a range between 1 and 139 months. One hundred and six patients (43%) had a follow-up time of more than 5 years. In 1.6% (*n* = 4) of all cases, residual cholesteatoma was found, located in the tympanic space and detected during MRI follow-up. Of these cases, the mean time to detection was 43 months post-operatively (range 34–52 months). No residual disease was found in the obliterated area. Recurrence formation of cholesteatoma was found in eight patients (3.2%). Concerning the Merchant classification, changes are depicted in Table 1. As is shown, surgery resulted in nearly 90% of cases in a dry ear. In 33 cases, revision surgery was needed for a variety of reasons. Eighteen revisions were performed because of hearing improvement (secondary ossicular chain reconstruction). The remaining 15 cases had a persistent draining ear which needed minor surgery (tympanic membrane closure, canalplasty, and removal of granulations). After revision surgery, a dry ear rate of 95% was achieved for the total cohort.

**Table 1:** Pre- and post-operative results of surgery

	Pre-operative *n* (%)	Post-operative *n* (%)	After revision *n* (%)
Merchant type			
0	16 (6.4)	164 (65.9)	182 (73.1)
1	11 (4.4)	59 (23.7)	55 (22.1)
2	49 (19.7)	22 (8.8)	11 (4.4)
3	173 (69.5)	4 (1.6)	0 (0)
Condition			
Dry ear	27 (10.8)	223 (89.6)	237 (95.2)
Wet ear	222 (89.2)	26 (10.4)	11 (4.4)

Merchant classification and dry ear rate. In total, 33 revisions were performed

### Hearing threshold

**Fig. 1:**
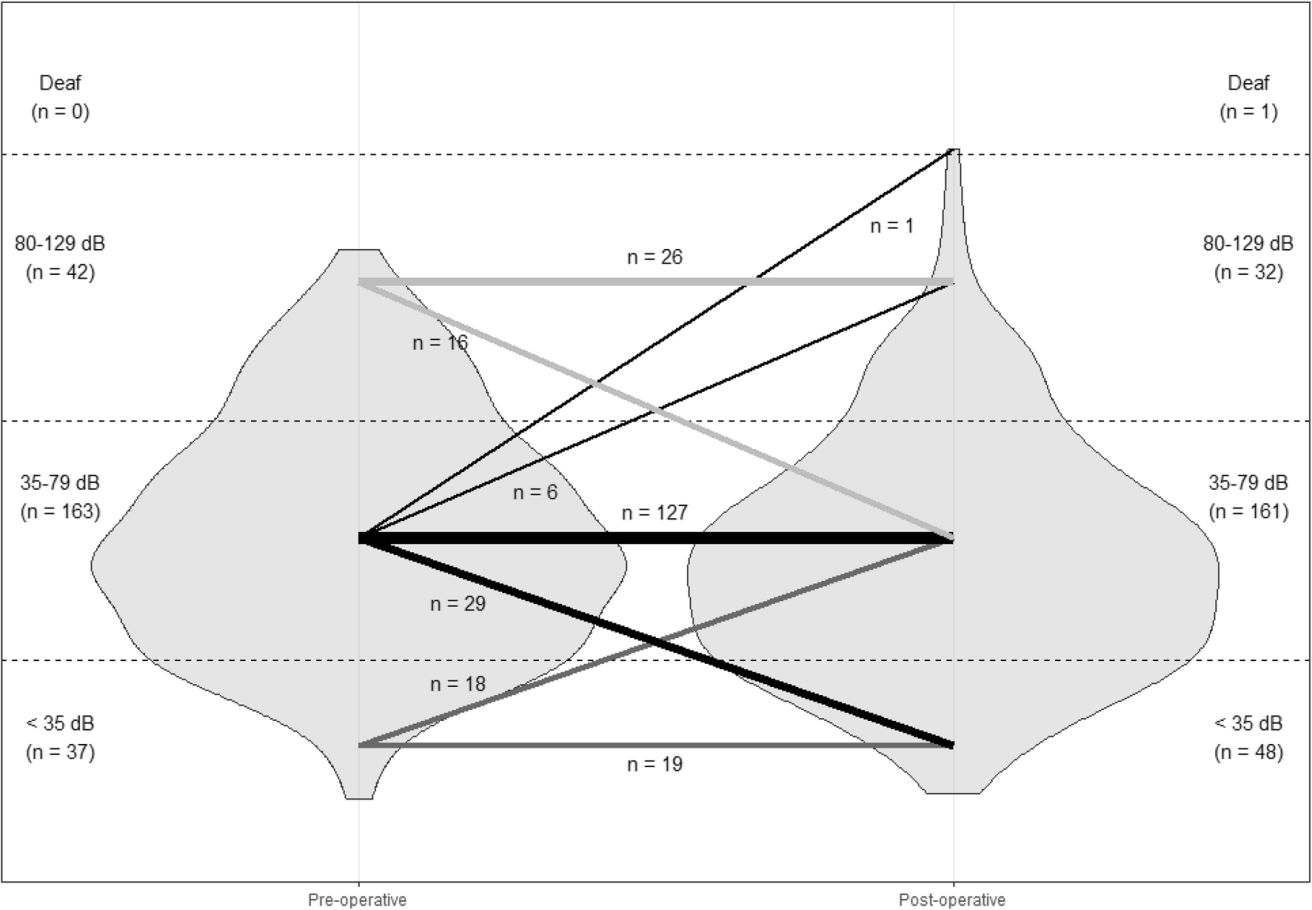
Violin plot of hearing thresholds. Hearing threshold in PTA (dB HL). Width of the violins depicts the amount of patients at that hearing threshold

**Fig. 2:**
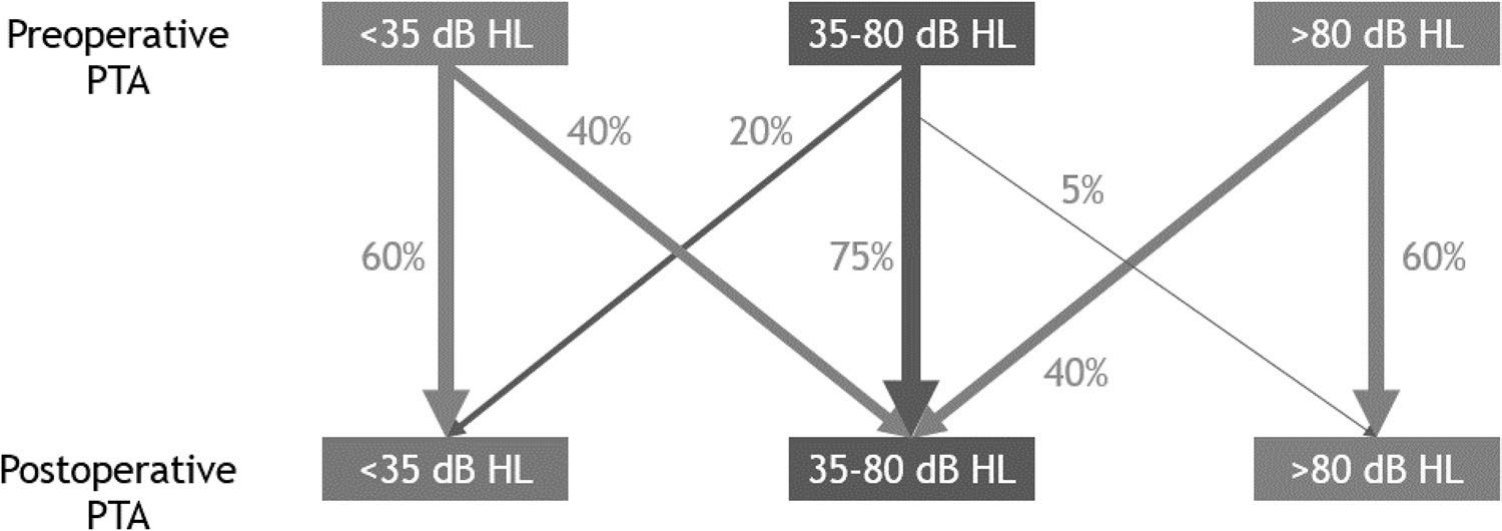
Pre-operative counselling diagram for expected post-operative hearing aid indication

Of 242 of 249 patients (97%), hearing data were complete and could be used for analysis. Pre- and post-operative PTAs of both air and bone conduction are shown in Table 2. We found a significant improvement in air conduction of 6 dB and a significant improvement of 1 dB in bone conduction levels. Twenty-eight cases (12%) had an improvement of more than 10 dB in bone conduction levels. Eight cases (3%) had a deterioration of bone conduction levels of more than 10 dB post-operatively (one case of a deaf ear and one case with severe high-frequency loss > 60 dB HL). The air-bone gap (ABG) was significantly reduced by 5 dB. The percentage of CHA indications did not differ after surgery (67% both pre- and post-operatively). Regarding the individual changes, a population shift in the violin plot (Fig. 1**)** towards better hearing (i.e., a downward shift) is observed. A total of 29 patients (12%) with an indication for a CHA did not need a CHA post-operatively because of improved hearing level (< 35 dB HL). Sixteen patients (7%) who had no indication for a CHA pre-operatively because of hearing > 80 dB HL were able to get a CHA post-operatively because of improved hearing (35–79 dB HL). A counselling model for expected post-operative CHA indication was proposed derived from the changes in PTA pre- versus post-operatively (Fig. 2). All categories with ossicular replacement (PORP, TORP, and cartilage) showed a significant improvement in the mean PTA (Table 3). In total, 105 patients did not receive any form of ossicular chain reconstruction.

**Table 2:** Mean hearing thresholds of the total cohort.

	Pre-operative	Post-operative	*p* value
Air conduction PTA	57 dB HL	51 dB HL	< 0.001
Bone conduction PTA	22 dB HL	21 dB HL	0.04
Air bone gap	35 dB	30 dB	< 0.001
Indication for CHA	166 patients (67%)	162 patients (65%)	0.78

A PTA > 34 dB HL and < 81 dB HL is defined as an indication for CHA

**Table 3:** Hearing thresholds of the different ossicular chain reconstructions performed.

Type of ossicular reconstruction	Pre-operative PTA median (range); in dB HL	Post-operative PTA median (range); in dB HL	Difference (dB)	*p* value
None (*n* = 105)	54 (8–111)	55 (9–121)	1	0.45
PORP (*n* = 70)	51 (14–103)	43 (15–130)	8	0.01
TORP (*n* = 34)	66 (35–101)	46 (18–94)	20	< 0.001
Cartilage (40)	64 (34–96)	61 (26–98)	3	0.02

Air conduction levels are depicted

### Hearing rehabilitation

**Fig. 3:**
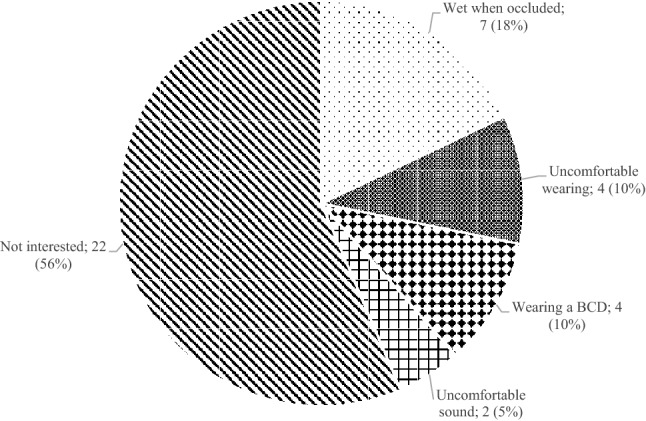
Reasons for non-usage of a CHA post-operatively

We included 106 patients for analysis of hearing rehabilitation as they had both an indication for CHA before and after surgery and a complete data set. Clear differences were seen when comparing pre-operative and post-operative CHA usage (Table 4). Nearly 60% of patients used a CHA post-operatively without any problems in comparison to 3% pre-operatively. A reduction was present of patients having problems of occluding their ear with a CHA. Around 40% of patients still did not use CHA despite a possible benefit post-operatively. A wide variety of reasons for this non-usage was given, as shown in Fig. 3. Of the group not interested in the use of CHA post-operatively, 91% (*n* = 20) of cases demonstrated a contralateral normal hearing ear (e.a. PTA < 35 dB HL). Only 7 (18%) cases did not wear a CHA due to occlusion problems post-operatively.

**Table 4:** Usage of CHA in patients with both pre- and post-operatively an indication for CHA

Usage of CHA	Pre-operative *n* (%)	Post-operative *n* (%)	*p* value
No	73 (69)	39 (38)	< 0.001
Yes, with occlusion problems	30 (28)	4 (4)	< 0.001
Yes, without any problems	3 (3)	63 (59)	< 0.001

We plotted the final hearing rehabilitation solutions against bilateral hearing thresholds to evaluate a possible predictive value of the PTA of the contralateral ear. It appeared that the contralateral hearing threshold does influence the choice for wearing a hearing aid on the operated ear, the contralateral ear, or bilaterally. However, cluster analysis did not support our hypothesised predictive models.

Of the 249 patients in this cohort, 22 patients wore BCD pre-operatively due to the troublesome cavity. Post-operatively, 9 (41%) patients still preferred a BCD over a CHA, whereas 13 patients (59%) preferred using a CHA.

## Discussion

Instable CWDMCs have been reported in 10 up to 40% of all cases [1, 5]. We report our outcomes of revision surgery including obliteration of the mastoid bowl of troublesome CWDMCs. High rates of dry, stable, and disease-free ears were achieved with comparable results to those reported in the literature [14–16]. No residual disease was found in the obliterated area during MRI follow-up. We strongly advocate long-time follow-up with MRI to detect residual disease after obliteration. Our results strengthen the hypothesis that revision surgery and obliteration of troublesome CWDMCs is the preferred treatment modality for troublesome CWDMCs [17]. Despite the fact that we only reported on revision surgery, the surgical technique can also be used in rare primary cases in which the posterior ear canal needs to be removed during surgery. Yung et al. published earlier about this kind of surgery in both primary and revision cases [8]. Like every operation, revision surgery with obliteration has its learning curve. We presented all patients who were operated using this technique in our centre, so a learning curve is included in our data. Reduction in operating time and complication rate was seen after 20 patients (unpublished data). Vartiainen et al. showed that more experience is important in reduction of revision surgery rate and healing time in otological surgery [18]. Stankovic et al. confirmed these findings when they compared the early and late surgical experience of the same surgeon [19].

It remains debatable whether hearing thresholds of canal wall down mastoidectomy are worse when compared to canal wall up cases [20]. Ossiculoplasties and revision surgery in canal wall down status have shown to have unfavorable results when compared with canal wall up status [21, 22]. Although hearing thresholds improved significantly in our investigated cohort, they are small and are, therefore, considered clinically irrelevant. This is in concordance with the previous literature demonstrating that hearing loss (mostly of a mixed type) remains present after such surgery [12, 16]. We observed a significant improvement of the various types of ossicular chain replacements used. This finding shows that performing ossiculoplasty is advised even in these small middle ear spaces. Perhaps, the aeration of the middle ear space is restored/improved after chronic inflammation ceases and, therefore, results in a better hearing threshold. The usually reported superiority of PORP over TORP could not be supported [23]. A possible explanation could be that the middle ear space is still limited after surgery and the eardrum often retracts in these troublesome cases making any type of reconstruction more difficult. Perhaps, stapes fixation is more present in troublesome cavities due to prolonged inflammation. As these data were not present in our retrospective study cohort, we were not able to evaluate the hearing results in those patients without a bad middle ear status and/or fixed stapes. These details could add valuable information to pre-operative counselling in managing expectations on post-operative hearing.

Although hearing thresholds improved for the group as a whole, we also demonstrate that the group eligible for hearing rehabilitation remains nearly unchanged. In addition, one should note that some individuals demonstrate a deterioration in hearing thresholds and, therefore, the need for hearing rehabilitation remains pivotal.

Troublesome cavities are likely to result in a difficulty to wear CHAs. Other factors such as: a large post-operative meatus, altered resonance frequency of the ear canal, and inadequate aeration due to occlusion all complicate CHA usage in CWDMCs [12, 24]. One would expect that this topic would have been investigated thoroughly. Yet, it seems that, to date, only one report describing 20 cases descriptively is available [12]. From this manuscript, it was concluded that revision surgery including obliteration of the mastoid bowl of troublesome CWDMCs makes CHA usage feasible and generally well tolerated. The potential benefit of enabling CHA rehabilitation after this type of surgery was mentioned by others but not investigated [14, 16]. Our large cohort demonstrates a significant increase of CHA usage and tolerance after surgery. We also believe that contralateral hearing threshold should be considered when evaluating CHA usage. Therefore, it should be considered in pre-operative counselling.

We found a slight shift in BCD rehabilitation toward CHA. Our data suggest that one should consider to primarily perform revision surgery in troublesome cavities to enable CHA usage before advising BCD. We potentially introduce an inclusion bias as we performed our analysis regarding CHA usage and tolerance in a selected subgroup of the total cohort as this subgroup of patients had a complete data set. We still feel that this subgroup is representable of the whole group. In our center, a CHA is the favored device for hearing rehabilitation due to lower costs and no need for further intervention. However, some have advocated a BCD in large ABGs to perform better in case of speech recognition [25, 26]. Combination of SubTotal Petrosectomy (STP) with a BCD could, therefore, be another option in chronically draining CWDMCs [27]. Unfortunately, we often see that the operated ear has worse bone conduction level compared to the contralateral ear. Sound from a BCD would be heard in the contralateral ear which results in only contralateral hearing. Providing in the possibility to wear a CHA can result in bilateral hearing in these cases.

For counselling of an individual patient, we now can present the chance of hearing threshold change in regard to CHA indication (within the Dutch reimbursement system) (Fig. 2). One should be aware of managing expectations of post-operative hearing as only 12% of the patients were operated out of the CHA indication group. We also tried to make a pre-operative model using contralateral hearing threshold to predict the final hearing rehabilitation outcome of the operated ear, but cluster analysis of our data unfortunately did not support a viable model. It seems that the choice to use a CHA is dependent on more factors than hearing threshold alone.

## Conclusion

Our study supports and strengthens the hypothesis that revision surgery and obliteration of CWDMCs enables successful CHA rehabilitation post-operatively. Hearing threshold improves significantly after such surgery, but the need for hearing rehabilitation remains necessary in most cases. Performing ossiculoplasty in troublesome CWDMCs seems to be beneficial. We supply a counselling diagram of the expected need for CHA rehabilitation postoperatively.
